# Whole exome sequencing revealed a novel homozygous variant in the DGKE catalytic domain: a case report of familial hemolytic uremic syndrome

**DOI:** 10.1186/s12881-020-01097-9

**Published:** 2020-08-24

**Authors:** Soraya Gholizad-kolveiri, Nakysa Hooman, Rasoul Alizadeh, Rozita Hoseini, Hasan Otukesh, Saeed Talebi, Mansoureh Akouchekian

**Affiliations:** 1 Department of Medical Genetics and Molecular Biology, school of medicine, Iran University of Medical Sciences, P.O.Box: 1449614525, Tehran, Iran; 2 Department of Pediatric Nephrology, Ali-Asghar Children Hospital, Iran University of Medical Sciences, Tehran, Iran

**Keywords:** Hemolytic-uremic syndrome, *DGKE*, Protein computational analysis, Whole exome sequencing

## Abstract

**Background:**

Atypical hemolytic uremic syndrome (aHUS) is a rare disease characterized by microangiopathic hemolytic anemia caused by small vessel thrombosis, thrombocytopenia, and renal failure. The common cause of aHUS is a dysregulation in the alternative complement pathway. Mutations in none complement genes such as diacylglycerol kinase epsilon (*DGKE*) can also result in this syndrome.

**Case presentation:**

Here, we report on a 19-year-old female with the clinical diagnosis of aHUS, who has unaffected consanguineous parents and an older sibling who was deceased from aHUS when she was seven months old.

We performed whole exome sequencing (WES) followed by evaluation of detected variants for functional significance, using several online prediction tools. Next, in order to confirm the detected pathogenic variant in proband and segregation analysis in her family, Sanger sequencing was done. The novel variant was analyzed in terms of its impact on the protein 3-dimensional structure by computational structural modeling.

The results revealed that the proband carried a novel homozygous missense variant in *DGKE* located in exon 6 of the gene (NM_003647.3, c.942C > G [p.Asn314Lys]), and in silico analysis anticipated it as damaging. Protein computational study confirmed the influence of potential pathogenic variant on structural stability and protein function.

**Conclusion:**

We suggest that some variations in the catalytic domain of DGKE like p.Asn314Lys which can cause alterations in secondary and 3-D structure of protein, might lead to aHUS.

## Background

Thrombotic microangiopathies (TMA) are a spectrum of disorders which are characterized by hemolytic anemia, low platelets, and organ damage [1].

Thrombotic thrombocytopenic purpura (TTP) and hemolytic uremic syndrome (HUS) are two types of TMA which can be differentiated by measuring ADAMTS13 (a disintegrin and metalloproteinase with a thrombospondin type1 motif, member13 or von Willebrand factor-cleaving protease) activity, which in case of TTP, is less than 10% [2, 3].

HUS is a rare disease characterized by the triad of microangiopathic hemolytic anemia, thrombocytopenia, and kidney failure. The most common cause of HUS in children is bacterial Shiga-like toxin produced e.g. by *Escherichia coli. (E. coli*) O: 157 and O: 104 serotypes [4] and the HUS induced by that is known as STEC-HUS (Shiga toxin-producing E.coli-HUS) or typical HUS [4, 5].

Non-bacterial HUS, which is caused by environmental and genetic factors is called atypical HUS (aHUS) and has a more severe clinical course in comparison with typical HUS. A high risk of developing end-stage renal disease (ESRD) is associated with frequent recurrences of aHUS [2]. aHUS is the result of genetic or autoimmune defects in the alternative pathway of complement which leads to overactivation and dysregulation of complement [5].

Various mutations in different genes that are associated with aHUS have been documented, which include: loss-of-function mutations in regulatory proteins of complement [complement factor H (*CFH),* complement factor I (*CFI),* membrane cofactor protein (*MCP/CD46)*] and gain-of-function mutations in promoting proteins of complement [complement component 3 (*C3),* complement factor B (*CFB)*] [3]. Recently, it has been identified that mutation in non-complement genes such as diacylglycerol kinase epsilon *(DGKE)* [5, 6], thrombomodulin (*THBD*) [6, 7], inverted formin 2 (*INF2)* [7] and methylmalonic aciduria and homocystinuria cobalamin C (cbLC) type (*MMACHC)* can also trigger aHUS [6, 7].

Whole Exome Sequencing (WES) is a genomic technique designed for the detection of genetic variations in all coding exons. In this study, we studied an Iranian family with a proband diagnosed with aHUS in infancy.

## Case presentation

### Clinical findings

**Fig. 1:**
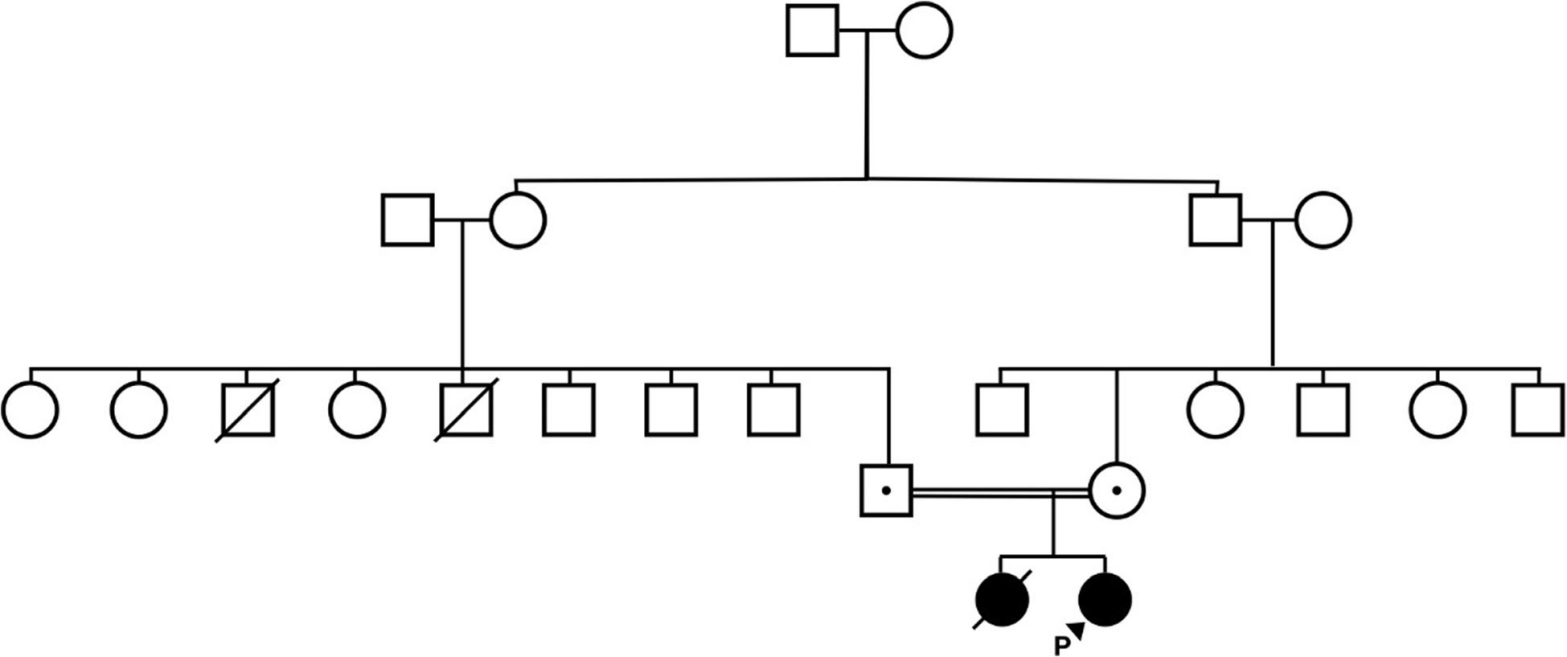
Pedigree of the Iranian family with familial aHUS. The proband is indicated with an arrow

In this research we studied an Iranian family with unaffected consanguineous parents who had two affected children. The older was deceased because of aHUS in 7 months of age, and the proband was a 19-year-old girl with chronic kidney disease, persistent hypertension, proteinuria and hematuria. There were no prominent genetic and metabolic disorders in family history (Fig. 1). The patient was referred to hospital at the age of 7.5 months with anuria, elevated creatinine and blood urea nitrogen following Shiga toxin independent gastroenteritis. Laboratory examination revealed that microangiopathic anemia and thrombocytopenia existed. Moreover, the presence of schistocyte and burr cell in blood smear was reported. Autoantibodies testing including antinuclear antibody (ANA), anti-DNA, Cytoplasmic antineutrophil cytoplasmic antibody(CANCA), perinuclear anti-neutrophil cytoplasmic antibody (PANCA), and anti ADAMTS13 antibody were negative. ADAMTS13 activity was > 10% and the plasma level of complement factors including CFH, CFI, and CFB was normal. All histopathological findings were consistent with the hemolytic uremic syndrome.

This research is reviewed and approved by the constituted Ethics Committee of Iran University of Medical Sciences (Tehran, Iran). We collected peripheral blood samples from the proband and her parents after genetic counseling and obtaining informed consent for molecular investigation.

### Whole exome sequencing

Genomic DNA was extracted using KBC blood DNA extraction kit (kawsar biotech company, Cat. No. K1135). After determination of concentration using NanoDrop, the patient’s genomic DNA was sent to Novogene, Hong Kong, for whole exome sequencing. In library preparation step, sequencing libraries of 150 bp were prepared using SureSelect Human All Exon kit (Agilent Technologies, CA, USA) followed by sequencing on Illumina paired-end high throughput sequencing (HiSeq 2000) platform (Illumina Inc., San Diego, CA, USA). Selected variants in the proband and her parents were validated by Sanger sequencing.

### In silico pathogenicity assessment of variants

Sequenced reads were aligned to NCBI human reference genome GRCh37 (hg19) and variants were called using biomedical genomics workbench v 5.0.2 (CLC BIO, Qiagen) (https://www.qiagenbioinformatics.com/products/clc-genomics-workbench/). The obtained variants were annotated using wANNOVAR (http://wannovar.wglab.org/) [8]. Then, the variants were filtered according to their frequency in population databases including the Genome Aggregation Database (gnomAD) (http://www.gnomad.broadinstitute.org), the 1000 Genome Project database (http://www.1000genomes.org), the single nucleotide polymorphism database (dbSNP) (http://www.ncbi.nlm.nih.gov/SNP/), the Exome Sequencing Project (ESP6500) (http://evs.gs.washington.edu), and Exome Aggregation Consortium (ExAC) Cambridge, MA; (http://exac.broadinstitute.org). Variants with minor allele frequency exceeding 0.05, were removed.

The potential pathogenicity of the obtained variants was predicted through several online prediction tools such as, Sorting Intolerant From Tolerant (SIFT) (http://sift.bii.a-star.edu.sg) [9], Combined Annotation Dependent Depletion (CADD)(https://cadd.gs.washington.edu/) [10],MutationTaster (http://www.mutationtaster.org/) [11], Polymorphism Phenotyping v2(PolyPhen-2) (http://genetics.bwh.harvard.edu/pph2/) [12], PROVEAN (Protein Variation Effect Analyzer) (http://provean.jcvi.org/) [13], and HOPE-CMBI (Have (y) Our Protein Explained) [14] (www.cmbi.ru.nl/hope/). Finally, interpretation of the novel mutation pathogenicity was done using the joint consensus recommendation of the American College of Medical Genetics and Genomics guideline (ACMG) [15] and semiquantitative, hierarchical evidence-based rules for locus interpretation (Sherloc) [16].

### Protein computational analysis

We used phyre2 (protein homology/analogy recognition engine) (http://www.sbg.bio.ic.ac.uk/phyre2) online tool to predict the possible 3-D structure of DGKE protein (UniProt ID: P52429) [17]. Afterwards, the quality of the interested residue in the top model was assessed using Phyre investigator, an advanced facility of phyre 2 for in-depth analysis of model. (Additional file 1).

Energy minimization was made using Swiss-Pdbviewer 4.0.1 in order to reach the most stable folding of the protein structure [18]; followed by amino acid replacement through Chimera 1.8.1. H-bonds and distances between residues were investigated in the structure. Multiple amino acid sequence alignment was performed to determine the residue conservation by Clustal Omega program (https://www.ebi.ac.uk › Tools › msa › clustalw2) and DGKE amino acid sequences of multiple species obtained from UniProt website (http://www.uniprot.org).

### Genetic findings

The exome analysis revealed 52,889 variants, including SNVs (single nucleotide variantions) and indels (Table 1). All obtained variants were filtered as described above, followed by an assessment using prediction software to identify variants with pathogenicity potential. Finally, filtered variants were sorted according to zygosity and CADD-PHRED score (cut-off =10) while focusing on the aHUS related genes. The only found homozygous variant was in *DGKE* which was a novel missense located in exon 6 of the gene (NM_003647.3, c.942C > G [p.Asn314Lys]). This variant has not been reported previously in ESP, 1000 Genomes Project, EXAC, dbSNP, and gnomAD. Mutation taster, polyphen2, SIFT, and PROVEAN prediction revealed that this variant was disease-causing, deleterious and damaging. The CADD-PHRED score was 17.08. According to ACMG and Sherloc criteria, the mentioned variant was likely pathogenic and pathogenic respectively. Sanger sequencing validated the variant as homozygous in proband and heterozygous in her unaffected parents (Additional file 2.)

**Table 1:** Summary of variants revealed via whole exome sequencing

Variant types	Proband
Total number of variants obtained	52,889
Total Indel variants	237
Exonic nonsynonymous variant^a^	754
Exonic nonsynonymous homozygous variant^a^	87
Frame shift^a^	2
Stop gain^a^	3
Inframe deletion^a^	1
Splice site^a^	1

^a^ minor allele frequency < 0.05

### Protein computational findings

The crystal structure of the mutated B chain of sphingosine kinase 1 (*Homo sapiens*, PDB ID: 3VZD) was used as a template for homology modeling by Phyre2.

Residues 214–544 of the sequence aligned with the mentioned enzyme with 58% coverage and shared 17% identity.

**Fig. 2:**
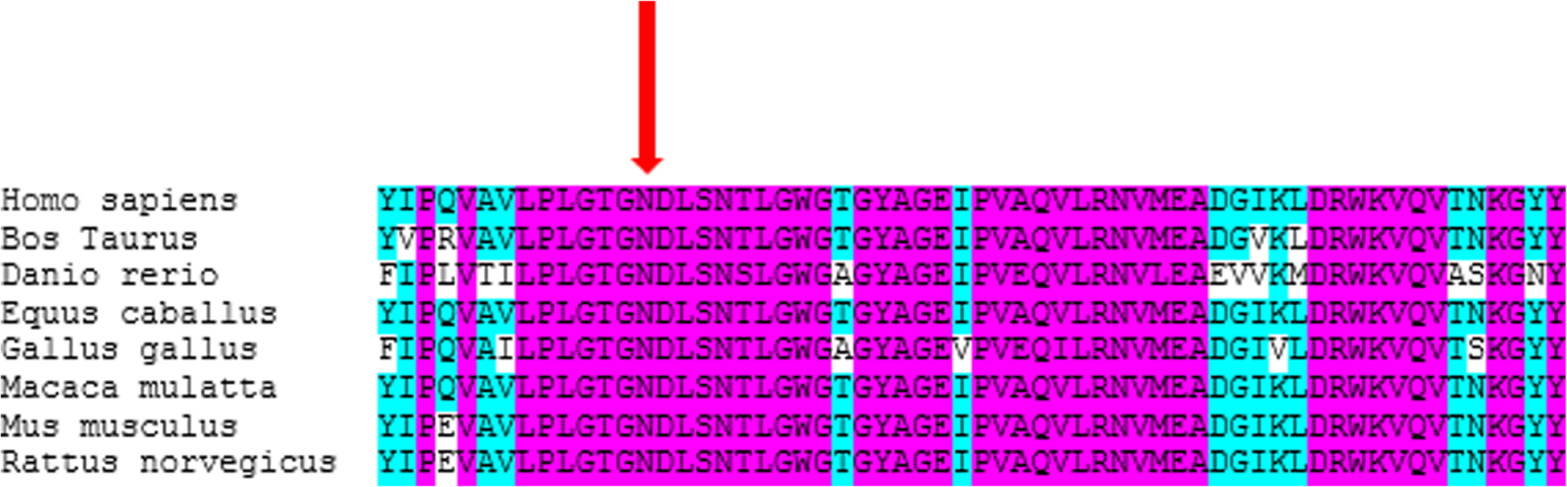
Amino acid alignment of DGKE protein amongst representative species around the site of p. Asn 314 (the mutated residue is displayed by red arrow)

**Fig. 3:**
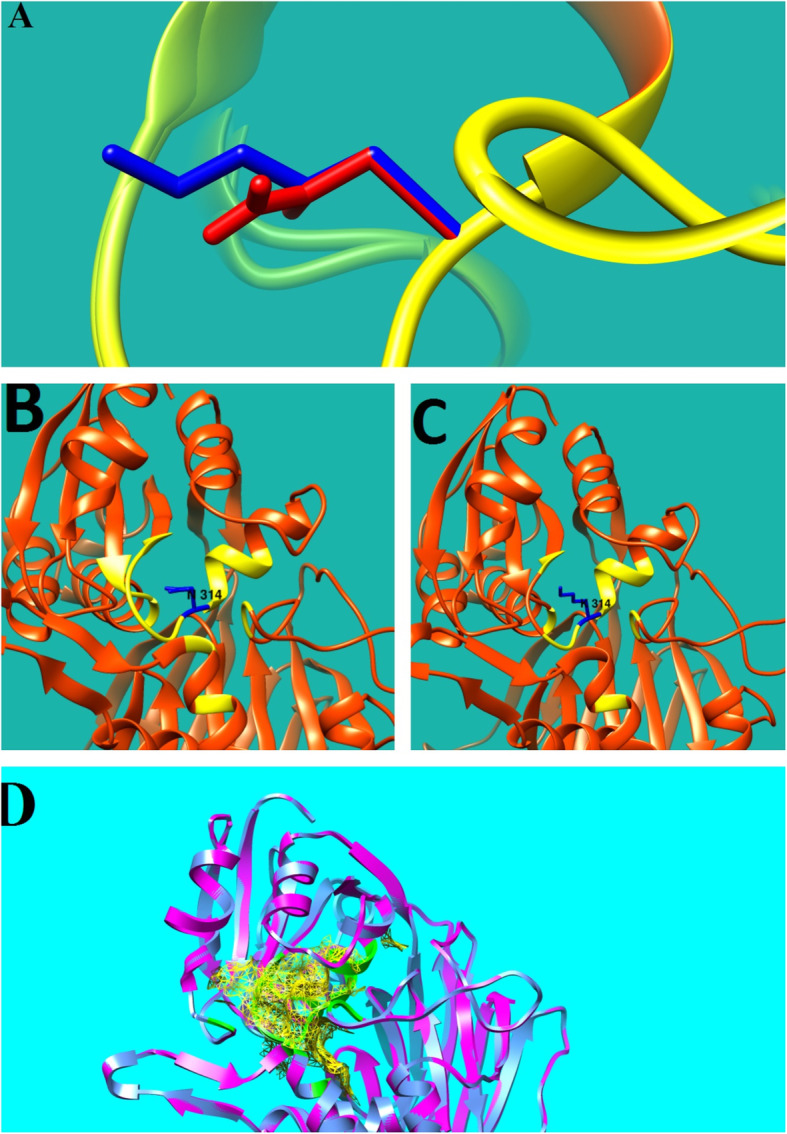
**a**. close up of A sn31 4L ys variant. The protein is colored yellow, the wild type and mutant residue are colored red and blue respectively, mutant residue is bigger than wild type. **b**. wild type residue (A sn) (blue), and its interaction with other residues among the protein structure (yellow). **c**. mutant residue (L ys) (blue) and its interaction with other residues (yellow). **d**. the yellow space shows the zone of wild type residue and the green ribbon represents the zone of mutated residue

Sequence alignment showed that the asparagine is located at a highly conserved region (Fig. 2). The novel variant in *DGKE* **(**c.942C > G) causes the asparagine to be replaced with lysine. After replacement with mutant residue, interactions between wild type and other residues significantly changed (Fig. 3 and additional file 3). Also interactions between the wild type residue (p.Asn314) and Gly311 and Asn318 leads to formation of two H-bonds while the mutant residue (p.Lys314) interacts with Ser317 and Asn318 and forms two H-bonds. (Additional file 4).

In addition, the amino acid substitution brings about an increase in the distance between Thr312 and Asp315 which can disturb their function as pocket amino acids (additional file 5).

### Homozygosity around the detected variant

All of the homozygous common variant were investigated in annotated VCF file, and a region greater than 11Mbp was detected on chromosome 17 around the NM_003647.3, c.942C > G [p.Asn314Lys] variant, in which all common variants were homozygous (from position 47,785,064 – to position 58,824,617, the detected variant position was 54,926,110).

## Discussion and conclusion

At least 10 diacylglycerol kinases (DGKs) are identified in mammals which phosphorylate diacylglycerols (DAGs) to phosphatidic acids (PAs). DGKE selectively phosphorylates arachidonic acid-containing DAG (arachidonoyl-DAG; AADAG), which is an intracellular signaling molecule in the endothelium, platelets, and podocytes. This protein has strong prothrombotic effects via protein kinase C activation. DGKE terminates AADAG signaling and prothrombotic activity by converting AADAG to phosphatidic acid. Therefore, TMA is expected in DGKE defects [5, 19].

In 2013, Lemaire and colleagues revealed several homozygous and compound heterozygous variants in *DGKE* which caused the autosomal recessive form of aHUS [20] .Affected subjects presented distinct clinical phenotypes: onset of aHUS before the age of one, occurrence of several relapses by the age of 5 and progressing to chronic kidney disease with age, continuous presence of hypertension, hematuria and proteinuria. Moreover, there was no evidence of complement activation in the vast majority of them [5, 19, 20]. The Iranian patient presented in this study had almost all of these clinical features*.*

The novel homozygous missense variant was not previously reported in population databases; however, another synonymous variant at the same position was reported with a low allele frequency. As mentioned above, this variant (c.942C > T, p.Asn314Lys) was predicted to be damaging, disease-causing and deleterious according to SIFT/PROVEAN, mutation taster and polyphen2 respectively, and the CADD-PHRED score was 17.08.

The catalytic domain of DGKE includes 141 amino acids (215 to 356) and the novel missense variant (c.942C > G, p.Asn314Lys), which is located in this domain, affects the main enzyme activity.

Unlike the wild type asparagine residue which is neutral, the mutant lysine residue has a positive charge and is also bigger. Hence, this alteration might result in excretion of the ligand or other residues with the same charge, and also bumps may be formed because of the bigger size of the mutant residue. As a result the catalytic domain may be disorganized and its function may be disturbed.

In addition, alterations in interactions with the surrounding amino acids after amino acid substitution, may influence the function of the protein (Fig. 3). Also, given that the wild type amino acid is located just before the initial part of the alpha helix, the changes in H-bonds can affect the protein secondary structure and subsequently influence the function (additional file 6). Furthermore, increase in the distance between two pocket amino acids which are adjacent to Lys314 may disturb their function as pocket amino acids.

The results of prediction software packages and 3-D structure analysis suggest that the *DGKE* p.Asn314Lys variant might have a potentially pathogenic effect on enzyme activity.

The segregation analysis revealed the autosomal recessive inheritance pattern of the variant. (Additional file 2).

According to the ClinVar database, 35 variants are identified in *DGKE* to date, of which 80% are pathogenic and likely pathogenic, and there are no benign or likely benign variants in the catalytic domain.

Based on ACMG guideline, the missense variant is categorized as a likely pathogenic variant for the following reasons: it does not exist in controls of population databases (Exome Sequencing Project, Exac and 1000 Genomes Project) (PM2), the prediction software packages suggested it to have a damaging effect on DGKE protein (PP3), clinical phenotype and family history were quite specific for aHUS (PP4) and the (c.942C > G, p.Asn314Lys) variant is located in the catalytic domain between two pocket amino acids which is crucial for the enzyme activity, and based on ClinVar database, there were no benign or likely benign variants in the catalytic domain (PM1). According to the Sherloc criteria, the variant is categorized as pathogenic, due to being absent in population databases (1P), being predicted as deleterious by protein prediction software packages (0.5P), and for being located on the critical region of the enzyme (4P). We could consider the (c.942C > G, p.Asn314Lys) variant in *DGKE* as a causative variant for aHUS; however, additional studies on the effects of this variant on DGKE 3-D structure would be helpful for collecting more evidence.

In view of the point that the inheritance pattern of the disorder is compatible with autosomal recessive, and the parents are first cousins - so their DNA is 12.5% identical and they will have a 12.5% chance of sharing two copies of rare pathogenic alleles or two identical by descent (IDB) alleles from a common ancestor [21]- we investigated whether the c.942C > G, p.Asn314Lys was located in a region of homozygosity. We detected a region of over 11Mbp on chromosome 17 in which all common variants are homozygous, and the c.942C > G, p.Asn314Lys variant belongs to this region of homozygosity.

We can detect the region of homozygosity by whole exome sequencing data. Theis et al. [22] identified a novel homozygous disease gene using Linkage analysis, homozygosity mapping and whole exome sequencing as synergistic large scale genomic strategies. (Additional file 7) [22].

## Supplementary information

**Additional file 1. **quality assessment of the 3-D structure suggested by phyre2 (using Ramachandran plot and prosA) and the residue of interest (using phyre investigator). Phyre investigator results revealed: **Figure S1–1.** The residue of interest alignment;** Figure S1–2.** The residue located between two pocket amino acids;** Figure S1–3.** Proq2 quality score of the interested residue; **Figure S1–4.** right Ramachandran analysis of the residue of interest. Ramachandran plot analysis of the model: **Figure S1–4.** lef: percent of residues located in favored and allowed region. The Z-score of the model was determined by ProSA: **Figure S1–5.**

**Additional file 2: Figure S2.** Confirmation of variant using Sanger sequencing

**Additional file 3: Figure S3.** Interaction of residue located at position 314 with the surrounding residues. **A:** Wild Type residue. **B:** Mutant residue

**Additional file 4: Figure S4.** H-bonds formed by wild type and mutant residues. **Figure S4–1.** H-bonds formed by wild type residue. **Figure S4–2.** H-bonds formed by mutant residue.

**Additional file 5: **The distances between Thr 312 and Asp 315 (pocket amino acids located on the sides of position 314). **Figure S5A.** wild type residue. **Figure S5B.** mutant residue.

**Additional file 6: Figure S6.** The prediction of secondary structure of the model by phyre2.

**Additional file 7: Table S7.** A large region of homozygsity on chromosome 17 around the detected variant.

## Data Availability

The raw datasets generated and/or analysed during the current study are not publicly available in order to protect participant confidentiality. The data and materials are available from the corresponding author (MA) upon reasonable request. The dataset corresponding to the DGKE gene can be found in NCBI under the accession number ENSG00000153933. The dataset corresponding to the DGKE protein sequence can be found in UniProt under the UniProt ID: P52429. The crystal structure of the mutated B chain of sphingosine kinase 1can be found in Protein Data Bank (PDB) under the PDB ID: 3VZD. Also to analyze the patient’s data the followings links have been used: Human reference genome (GRCh37/hg19) (https://www.ncbi.nlm.nih.gov/ assembly/GCF_000001405.13/), wANNOVAR (http://wannovar.wglab.org/) Genome Aggregation Database (gnomAD) (http://www.gnomad.broadinstitute.org), 1000 Genome Project database (http://www.1000genomes.org), the single nucleotide polymorphism database (dbSNP) (http://www.ncbi.nlm.nih.gov/SNP/), the Exome Sequencing Project (ESP6500) (http://evs.gs.washington.edu), Exome Aggregation Consortium (ExAC) Cambridge, MA; (http://exac.broadinstitute.org), Sorting Intolerant From Tolerant (SIFT) (http://sift.bii.a-star.edu.sg), Combined Annotation Dependent Depletion (CADD) (https://cadd.gs.washington.edu/), Mutation Taster (http://www.mutationtaster.org/), Polymorphism Phenotyping v2(PolyPhen-2) (http://genetics.bwh.harvard.edu/pph2/), PROVEAN (Protein Variation Effect Analyzer)(http://provean.jcvi.org/), and HOPE-CMBI (Have (y) Our Protein Explained)(www.cmbi.ru.nl/hope/), phyre2 (protein homology/analogy recognition engine) (http://www.sbg.bio.ic.ac.uk/phyre2),Clustal Omega (https://www.ebi.ac.uk › Tools › msa › clustalw2), UniProt website (http://www.uniprot.org). The c.942C > G variation was submitted to ClinVar database (https://www.ncbi.nlm.nih.gov/clinvar/) and the ClinVar accession number SCV001142646 assigned to it.

## Abbreviations

HUS: Hemolytic uremic syndrome
aHUS: Atypical HUS
DGKE: Diacylglycerol kinase epsilon
WES: Whole exome sequencing
TMA: Thrombotic microangiopathies
TTP: Thrombotic thrombocytopenic purpura
ADAMTS13: A disintegrin and metalloproteinase with a thrombospondin type1 motif member13
*E. coli*: *Escherichia coli*
STEC-HUS: Shiga toxin-producing E.coli-HUS
ESRD: End-stage renal disease
CFH: Complement factor H
CFI: Complement factor I
MCP: Membrane cofactor protein
C3: Complement component 3
CFB: Complement factor B
THBD: Thrombomodulin
INF2: Inverted formin 2
MMACHC: Methylmalonic aciduria and homocystinuria cobalamin C (cbLC) type
ANA: antinuclear antibody
CANCA: Cytoplasmic antineutrophil cytoplasmic antibody
PANCA: perinuclear anti-neutrophil cytoplasmic antibody
gnomAD: Genome AGGREGATION DATABASE
dbSNP: Single nucleotide polymorphism database
ESP: Exome sequencing project
ExAC: Exome aggregation consortium
SIFT: Sorting intolerant from tolerant
CADD: Combined annotation dependent depletion
PolyPhen-2: Polymorphism phenotyping v2
PROVEAN: Protein variation effect analyzer
ACMG: American College of medical genetics and genomics guideline
Sherloc: Semiquantitative, hierarchical evidence-based rules for locus interpretation
phyre2: Protein homology/analogy recognition engine
SNV: Single nucleotide variantion
DAG: Diacylglycerol
DGK: Diacylglycerol kinase
AADAG: Arachidonic acid-containing DAG
PA: Phosphatidic acid
IDB: Identical by descent
