# Supplementary material for: Whole exome sequencing revealed a novel homozygous variant in the DGKE catalytic domain: a case report of familial hemolytic uremic syndrome
Source: BMC Med Genet. 2020 Aug 24;21:169. doi: 10.1186/s12881-020-01097-9 (PMC7446132; doi:10.1186/s12881-020-01097-9)
Supplement: Supplementary file 1 — Additional file 1. quality assessment of the 3-D structure suggested by phyre2 (using Ramachandran plot and prosA) and the residue of interest (using phyre investigator). Phyre investigator results revealed: Figure S1–1. The residue of interest alignment; Figure S1–2. The residue located between two pocket amino acids; Figure S1–3. Proq2 quality score of the interested residue; Figure S1–4. right Ramachandran analysis of the residue of interest. Ramachandran plot analysis of the model: Figure S1–4. lef: percent of residues located in favored and allowed region. The Z-score of the model was determined by ProSA: Figure S1–5. [file 12881_2020_1097_MOESM1_ESM.docx]

**quality assessment of the 3-D structure suggested by phyre2 (using Ramachandran plot and prosA) and the residue of interest ( using phyre investigator).**

The coverage alignment of the top model suggested by phyre2 (protein homology/analogy recognition engine) (<http://www.sbg.bio.ic.ac.uk/phyre2> ) was 58%.

phyre investigator (an advanced facility of phyre2) results revealed :

**A**: The residue of interest alignment confidence score was 0.00 (0=good, 1=bad) (figure S.1-1) and it was located between two pocket amino acids (figure S.1-2).

**B**: Proq2 (Protein Quality Predictors) quality score of the residue of interest was 0.272 (0=good, 1=bad) (figure S.1-3) (proQ2 (<http://proq2.wallnerlab.org/> ) is an algorithm to model quality assessment that predicts global and local quality of protein models) (21).

**C**: Ramachandran analysis of the residue of interest showed that it was in the favorable region (figure S.1-4right).

Also the model suggested by phyre2 was validated using Ramachandran plot (<http://mordred.bioc.cam.ac.uk/~rapper/rampage.php> ) (19)(figure S.1-4 left). Ramachandran plot revealed that 96.9% of residues were in the favored and allowed region

Then the Z-score of the model was determined by ProSA(protein structure analysis) (<https://prosa.services.came.sbg.ac.at/prosa.php> ) (18)(figure S.1-5).

According to the Z-score plot, the structure is within the range of Z-scores belong to all experimentally (X-ray or NMR) determined protein structure with similar size.


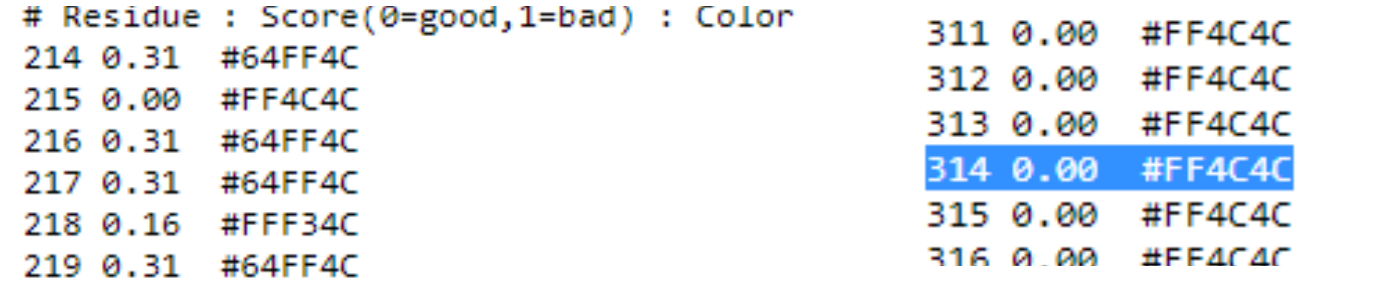


**Figure S.1-1**.alignment confidence score at phyre investigator


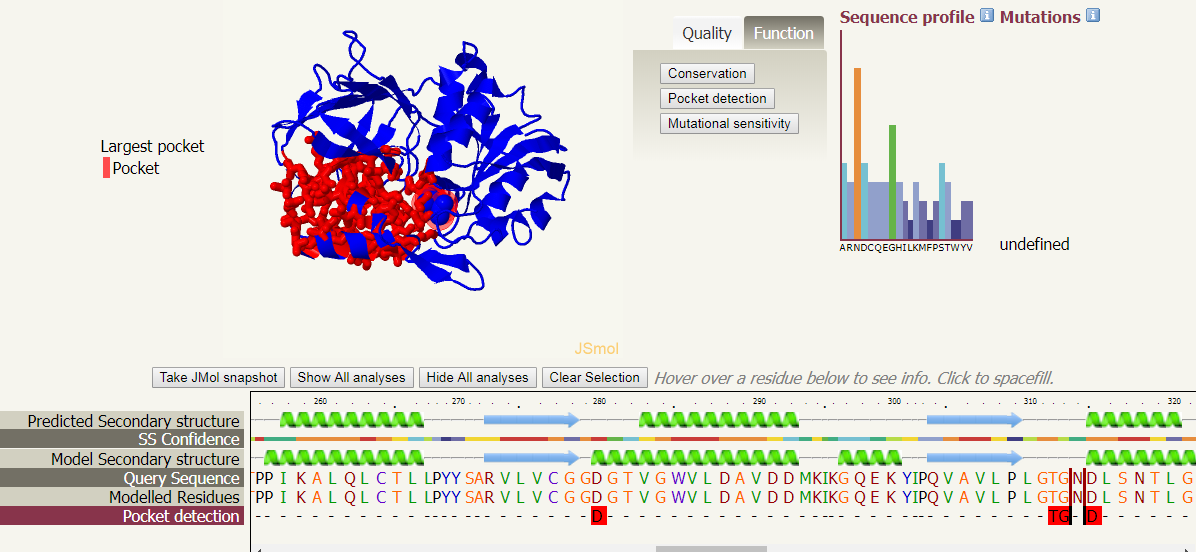


**Figure S.1-2.** phyre investigator reveals p.N314 was located between two pocket amino acid


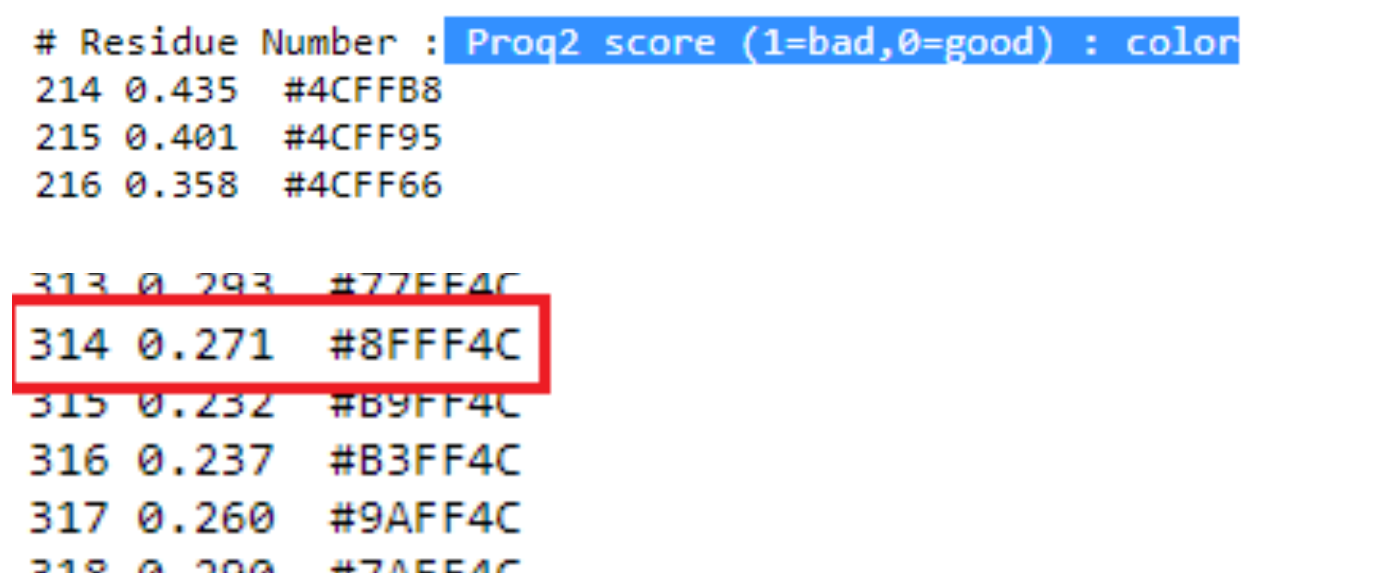


**Figure S.1-3.** phyre investigator reveals proQ2 score of the residue of interest was 0.2


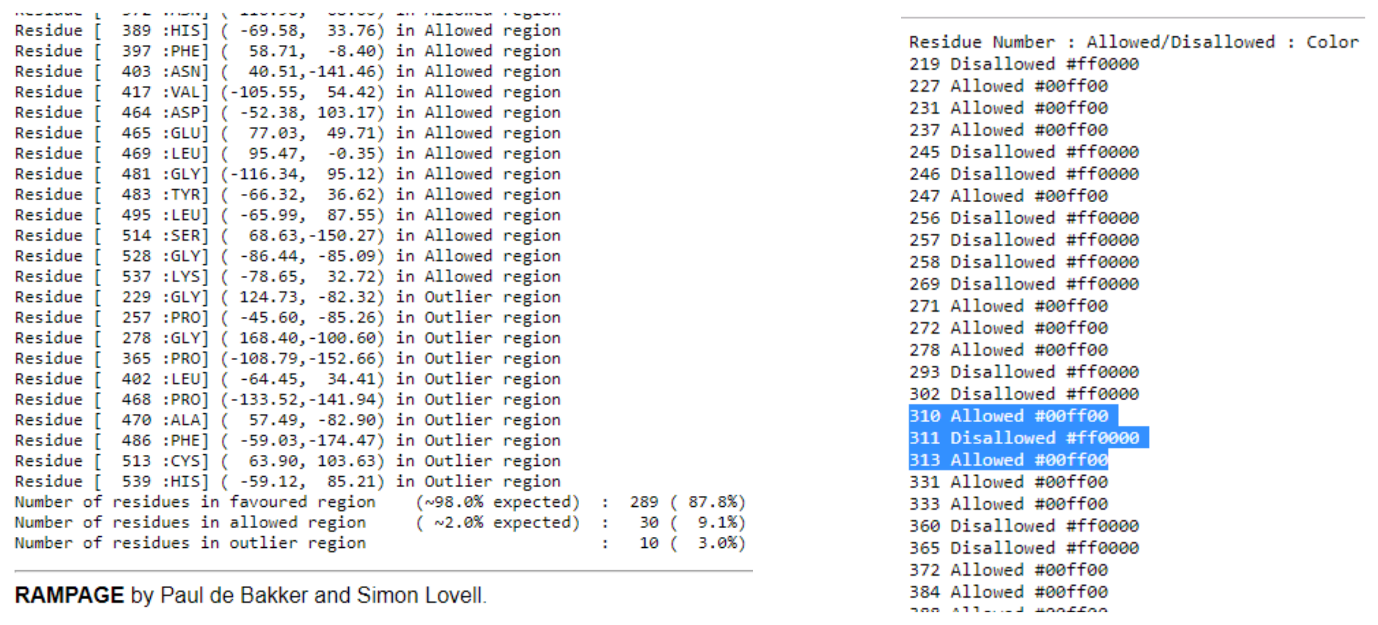


**Figure S.1-4 Right:** phyre investigator results revealed the residue of interest located at favored region. **Left:** Ramachandran plot analysis of the model revealed 96.9% of residues are in favored and allowed region.


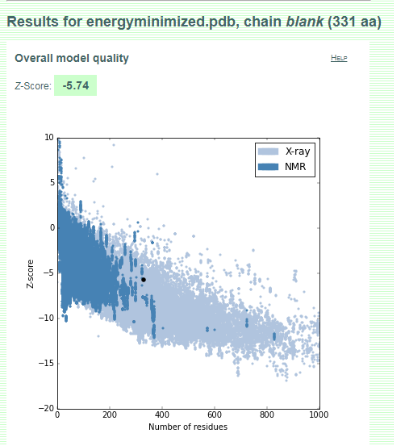


**Figure S.1-5:** prosA Z-score of the model.
