# Supplementary material for: Whole exome sequencing revealed a novel homozygous variant in the DGKE catalytic domain: a case report of familial hemolytic uremic syndrome
Source: BMC Med Genet. 2020 Aug 24;21:169. doi: 10.1186/s12881-020-01097-9 (PMC7446132; doi:10.1186/s12881-020-01097-9)
Supplement: Supplementary file 2 — Additional file 2: Figure S2. Confirmation of variant using Sanger sequencing [file 12881_2020_1097_MOESM2_ESM.docx]

**Confirmation of variant using Sanger sequencing**


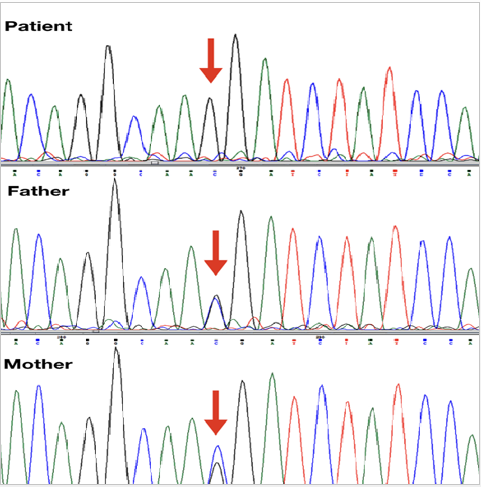


**Figure S.2**. sequencing chromatograms of the proband and her parents
