# Supplementary material for: Whole exome sequencing revealed a novel homozygous variant in the DGKE catalytic domain: a case report of familial hemolytic uremic syndrome
Source: BMC Med Genet. 2020 Aug 24;21:169. doi: 10.1186/s12881-020-01097-9 (PMC7446132; doi:10.1186/s12881-020-01097-9)
Supplement: Supplementary file 3 — Additional file 3: Figure S3. Interaction of residue located at position 314 with the surrounding residues. A: Wild Type residue. B: Mutant residue [file 12881_2020_1097_MOESM3_ESM.docx]

**Interaction of residue located at position 314 with the surrounding residues**


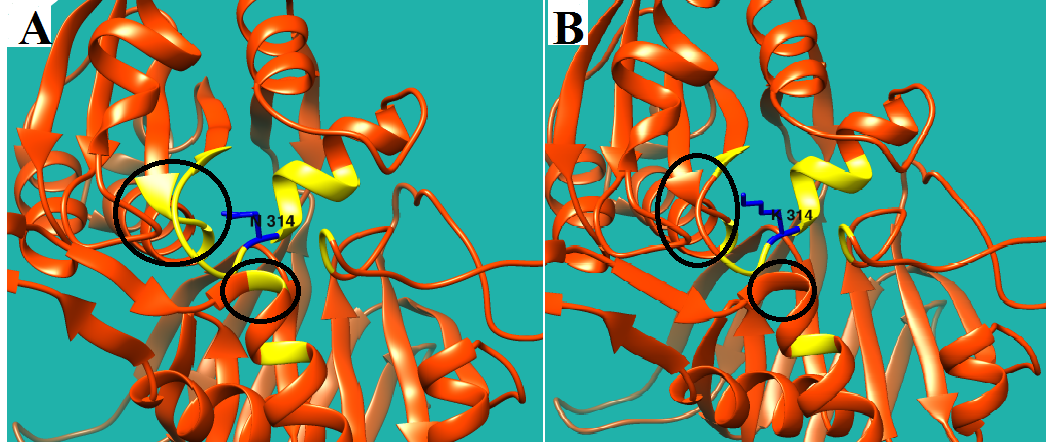


**Figure S.3.** The interaction of the residue located at position 314 with the surrounding residues is shown in yellow. **A**. Asparagine (Wild Type residue) and **B**. Lysine (mutant residue). The difference between the interaction of the wild type and the mutant residues is illustrated using black ellipses.
