# Supplementary material for: Whole exome sequencing revealed a novel homozygous variant in the DGKE catalytic domain: a case report of familial hemolytic uremic syndrome
Source: BMC Med Genet. 2020 Aug 24;21:169. doi: 10.1186/s12881-020-01097-9 (PMC7446132; doi:10.1186/s12881-020-01097-9)
Supplement: Supplementary file 4 — Additional file 4: Figure S4. H-bonds formed by wild type and mutant residues. Figure S4–1. H-bonds formed by wild type residue. Figure S4–2. H-bonds formed by mutant residue. [file 12881_2020_1097_MOESM4_ESM.docx]

**H-bonds formed by wild type and mutant residues**


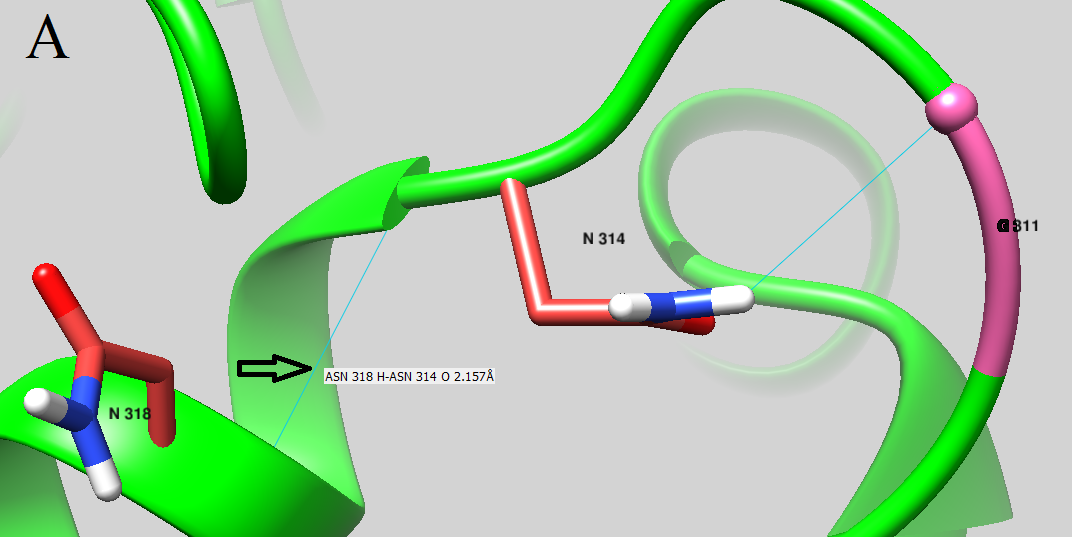


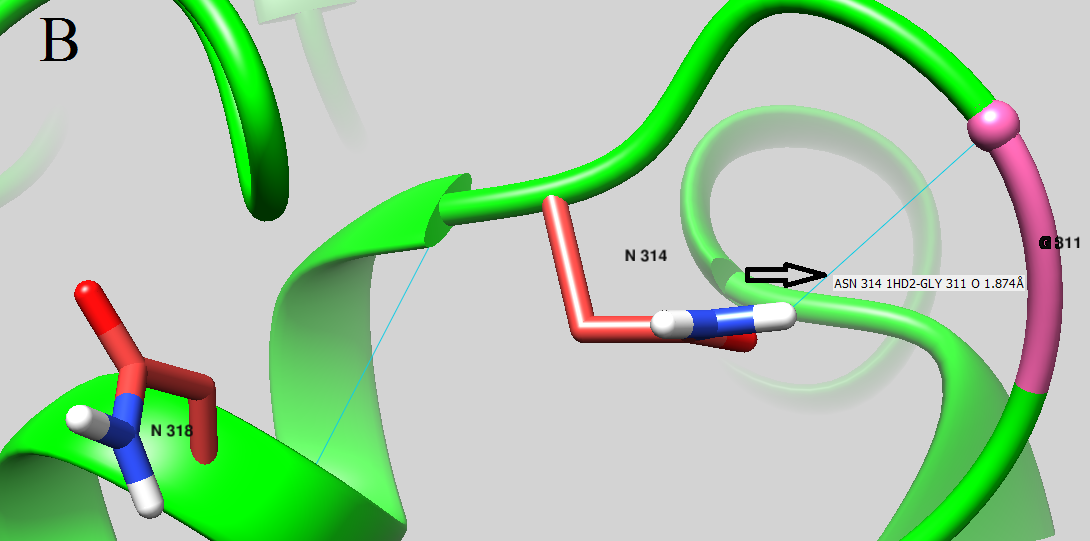


**Figure S.4-1. A**. H-bonds formed by Asn314 and Asn318 **B.** H-bonds formed by Asn314 and Gly311. Wild type residue formed H-bonds with two residues located at N-terminal (G311) and C-terminal (N318).


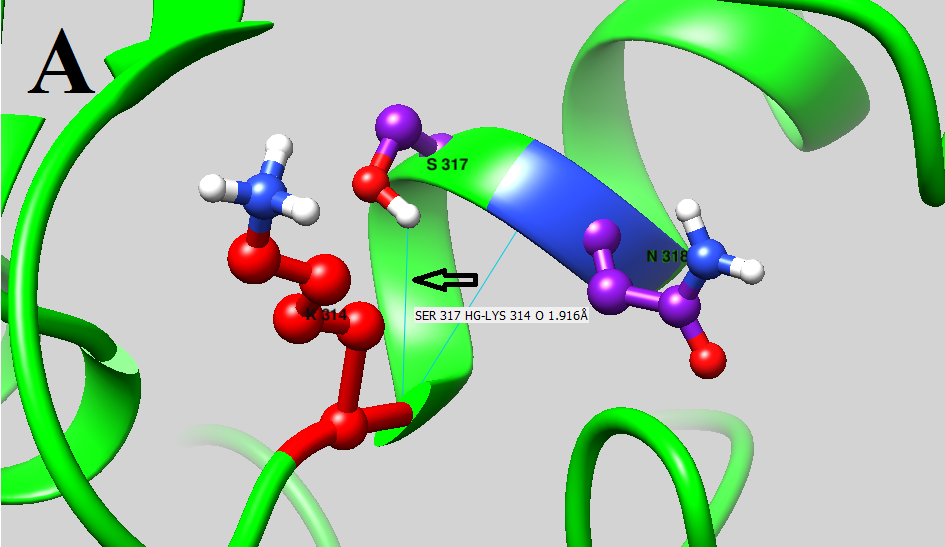


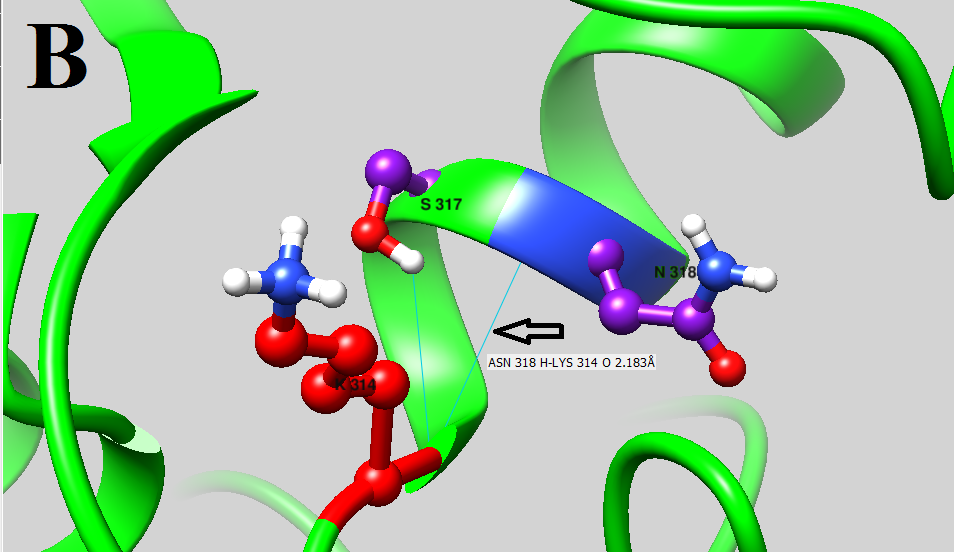


**Figure S.4-2 A.** H-bonds formed by Lys314 and Ser317 **B.** H-bond formed by Lys314 and Asn318. Mutant residue formed H-bonds with 2 residues located at C-terminal (S317 and N318).
