# Supplementary material for: Whole exome sequencing revealed a novel homozygous variant in the DGKE catalytic domain: a case report of familial hemolytic uremic syndrome
Source: BMC Med Genet. 2020 Aug 24;21:169. doi: 10.1186/s12881-020-01097-9 (PMC7446132; doi:10.1186/s12881-020-01097-9)
Supplement: Supplementary file 5 — Additional file 5: The distances between Thr 312 and Asp 315 (pocket amino acids located on the sides of position 314). Figure S5A. wild type residue. Figure S5B. mutant residue. [file 12881_2020_1097_MOESM5_ESM.docx]

**The distances between Thr 312 and Asp 315 (pocket amino acids located on the sides of position 314)**


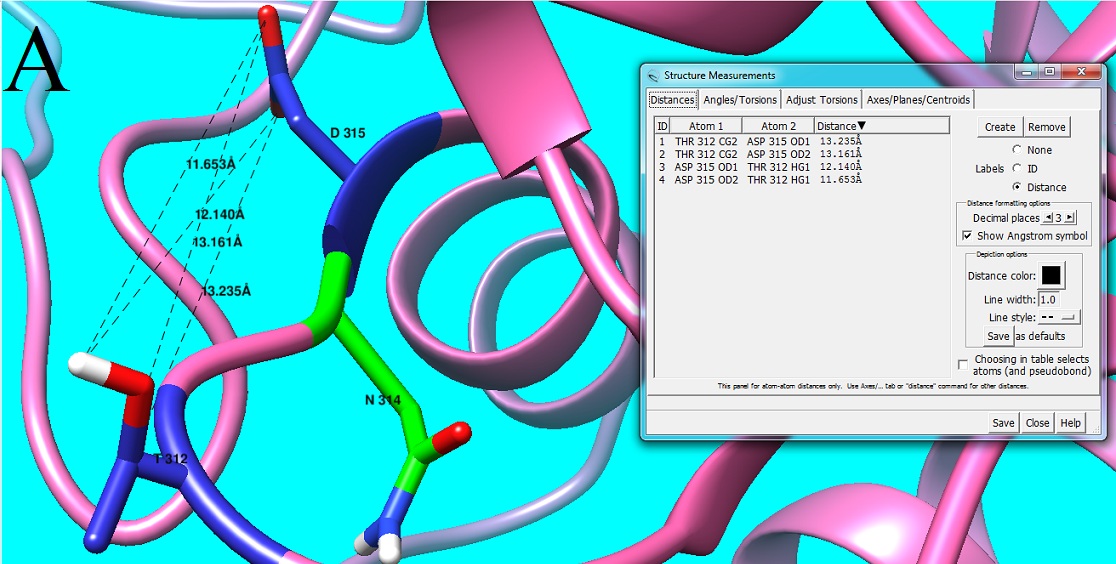


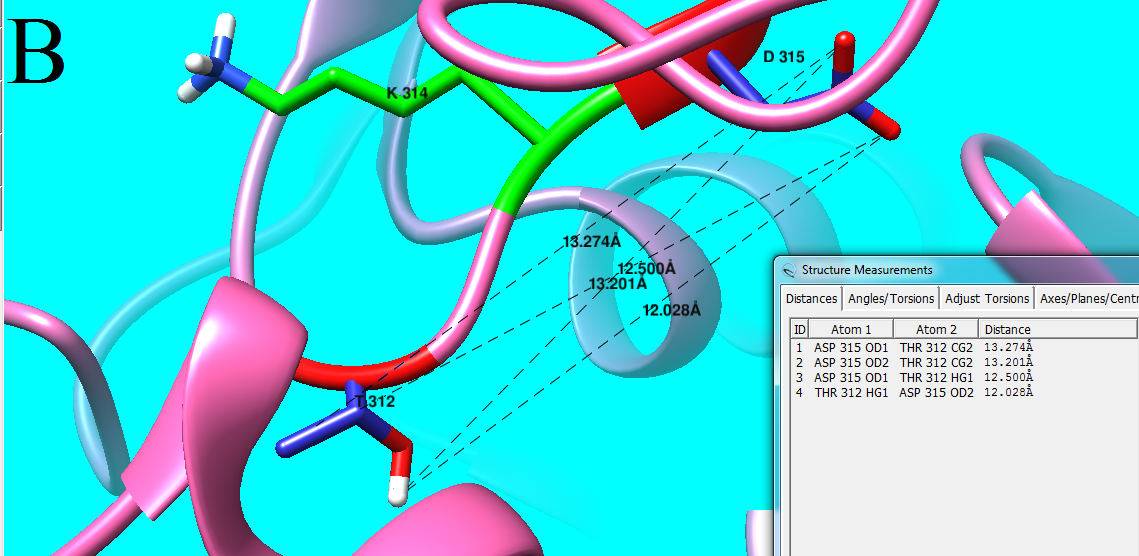


**Figure S.5.A.** Wild type residue (Asn) is located at position 314. **B.** Mutant residue (Lys) is located at position 314.

The distances, after replacement of asparagine with lysine, increased. As Thr312, Gly313 and Asp315 are pocket amino acids, increased distances may affect the interaction between enzyme and substrate.
