# Supplementary material for: Whole exome sequencing revealed a novel homozygous variant in the DGKE catalytic domain: a case report of familial hemolytic uremic syndrome
Source: BMC Med Genet. 2020 Aug 24;21:169. doi: 10.1186/s12881-020-01097-9 (PMC7446132; doi:10.1186/s12881-020-01097-9)
Supplement: Supplementary file 6 — Additional file 6: Figure S6. The prediction of secondary structure of the model by phyre2. [file 12881_2020_1097_MOESM6_ESM.docx]

**The prediction of secondary structure of the model by phyre2**


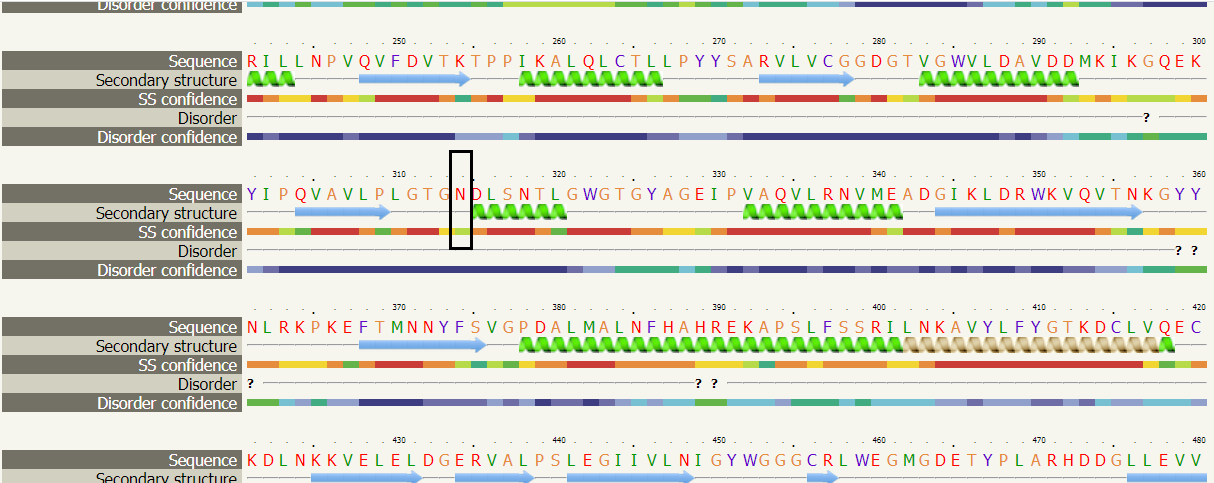


**Figure S.6**: The prediction of secondary structure of the model by phyre2 revealed that p.N314 is located just before alpha helix initiation site.
